# Functional Analysis of a Breast Cancer-Associated Mutation in the Intracellular Domain of the Metalloprotease ADAM12

**DOI:** 10.1371/journal.pone.0037628

**Published:** 2012-05-25

**Authors:** Dorte Stautz, Ulla M. Wewer, Marie Kveiborg

**Affiliations:** Department of Biomedical Sciences & Biotech Research and Innovation Centre, Copenhagen University, Ole Maaløes Vej, Copenhagen, Denmark; Wayne State University School of Medicine, United States of America

## Abstract

A recently identified breast cancer-associated mutation in the metalloprotease ADAM12 alters a potential dileucine trafficking signal, which could affect protein processing and cellular localization. ADAM12 belongs to the group of A Disintegrin And Metalloproteases (ADAMs), which are typically membrane-associated proteins involved in ectodomain shedding, cell-adhesion, and signaling. ADAM12 as well as several members of the ADAM family are over-expressed in various cancers, correlating with disease stage. Three breast cancer-associated somatic mutations were previously identified in ADAM12, and two of these, one in the metalloprotease domain and another in the disintegrin domain, were investigated and found to result in protein misfolding, retention in the secretory pathway, and failure of zymogen maturation. The third mutation, *p.L792F* in the ADAM12 cytoplasmic tail, was not investigated, but is potentially significant given its location within a di-leucine motif, which is recognized as a potential cellular trafficking signal. The present study was motivated both by the potential relevance of this documented mutation to cancer, as well as for determining the role of the di-leucine motif in ADAM12 trafficking. Expression of ADAM12 *p.L792F* in mammalian cells demonstrated quantitatively similar expression levels and zymogen maturation as wild-type (WT) ADAM12, as well as comparable cellular localizations. A cell surface biotinylation assay demonstrated that cell surface levels of ADAM12 WT and ADAM12 *p.L792F* were similar and that internalization of the mutant occurred at the same rate and extent as for ADAM12 WT. Moreover, functional analysis revealed no differences in cell proliferation or ectodomain shedding of epidermal growth factor (EGF), a known ADAM12 substrate between WT and mutant ADAM12. These data suggest that the ADAM12 *p.L792F* mutation is unlikely to be a driver (cancer causing)-mutation in breast cancer.

## Introduction

**Figure 1:**
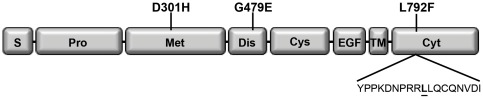
Schematic illustration of ADAM12 indicating the published breast cancer-associated mutations and the di-leucine motif in the cytoplasmic tail. Schematic illustration of the domain organization of ADAM12. S: Signal peptide; Pro: Prodomain; Met: Metalloprotease domain; Dis: Disintegrin domain; Cys: Cysteine-rich domain; EGF: EGF-like domain; TM: Transmembrane domain; Cyt: Cytoplasmic tail. Positions of the three breast cancer-associated mutations are indicated and the amino acid sequence containing the di-leucine motif is shown.

ADAM12 is a member of the ADAMs (A Disintegrin And Metalloproteases) family of transmembrane zinc-dependent proteases with a characteristic domain structure (Fig. 1). ADAMs are involved in regulating integrin-mediated cell adhesion, cell signaling and the proteolytic release, known as ectodomain shedding of cell surface-associated substrates [1]–[4]. Several members of the ADAM family are highly expressed in a variety of human carcinomas, likely contributing to tumor development and/or progression through the release of epidermal growth factor receptor EGFR ligands or effects on cell-cell or cell-matrix adhesion [1], [3]. We, and others previously showed that ADAM12 expression was markedly upregulated in different cancers [5]–[9], and that the level of ADAM12 in urine from breast and bladder cancer patients correlated with disease status and stage [10], [11]. ADAM12 promotes tumor progression in transgenic mouse models of breast and prostate cancer [6], [12], [13] and several ADAMs are considered as promising targets for cancer therapy [14]–[16]. Despite accumulating evidence for involvement of ADAMs in cancer, only a few cancer-related ADAM mutations have been reported (see ref. [17] for complete list). Human ADAM12 exists in two naturally occurring splice variants; ADAM12-L resembling the prototypical transmembrane ADAM protein shown in figure 1 and ADAM12-S, a soluble splice variant, lacking the transmembrane domain and the cytoplasmic tail. In an analysis of genomic changes in breast cancer, three somatic heterozygous mutations were found in ADAM12, *p.D301H* in the metalloprotease domain, *p.G479E* in the disintegrin domain, and *p.L792F* in the cytoplasmic domain (Fig. 1) [18]. Bioinformatic analysis predicted that only *p.D301H* and *p.G479E*, but not *p.L792F* were likely to be cancer-causing, since no changes were tolerated at these two positions [19]. Analysis of the *p.D301H* and *p.G479E* proteins strongly suggested misfolding of these mutants, since neither was secreted, both were retained in the endoplasmic reticulum (ER), and neither underwent zymogen maturation, a process mediated by furin that converts nascent 120-kDa ADAM12 to the mature 90-kDa form and occurs downstream of the ER [19]. The *p.L792F* mutation is situated in the second (from the N-terminus) of two di-leucine motifs in ADAM12. Although the *p.L792F* mutation was predicted to be inconsequential, it is of potential interest as it affects a di-leucine motif that is an important sorting signal in internalization and/or trafficking of several proteins (Fig. 1). Di-leucine motifs play critical roles in the sorting of many type I, type II, and multi-spanning transmembrane proteins, by associating with adaptor proteins, such as AP-complexes or Golgi-localized, γ-ear containing, ADP-ribosylation factor-binding proteins (GGAs) [20], [21].

We therefore sought to investigate if the *p.L792F* mutation in the cytoplasmic domain of ADAM12 (since only ADAM12-L is affected, the nomenclature ADAM12 is used for the sake of simplicity) altered protein processing, trafficking and internalization with potential functional implications for cell proliferation and protein ectodomain shedding. Rigorous analysis suggested that the ADAM12 *p.L792F* mutant was comparable to WT ADAM12 in every aspects analyzed, indicating that the di-leucine motif was not involved in the intracellular trafficking of ADAM12 and thus may not be contributing significantly to the breast cancer phenotype.

## Results and Discussion

### The ADAM12 wild-type and *p.L792F* mutant are similarly expressed and processed

**Figure 2:**
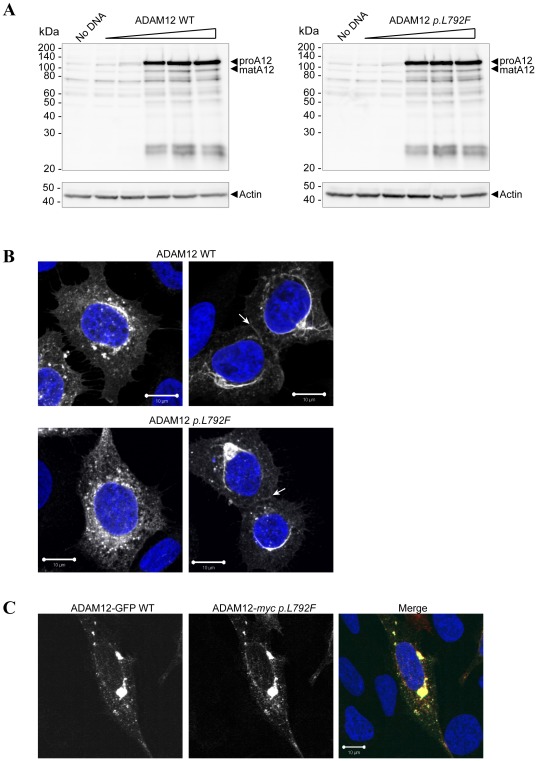
Expression and processing of ADAM12 wild-type and *p.L792F* mutant. (**A**) ADAM12 *p.L792F* is expressed and processed similarly to WT ADAM12. 293VnR cells were transiently transfected with increasing amounts of either ADAM12 WT or ADAM12 *p.L792F*. After 48 h cells were lysed, total protein amounts were determined and proteins analyzed by SDS-PAGE and subsequent western blot with an anti-ADAM12 antibody. The ADAM12 proform (proA12) is ∼120 kDa and the mature form (matA12) is ∼95 kDa (arrowheads). The membranes were re-probed with anti-actin antibody to confirm equal loading. (**B**) Similar immunofluorescent staining of ADAM12 WT and *p.L792F* mutant. 293VnR cells were transiently transfected with ADAM12 WT or ADAM12 *p.L792F* and after two days of incubation, seeded onto FBS coated coverslips. Cells were fixed in 4% PFA, permeabilized in Triton X-100, labeled with ADAM12 monoclonal antibody 6E6 for 1 hour, and followed by staining with secondary antibody and DAPI. Two representative images acquired using confocal laser-scanning microscopy are shown for each protein. White arrows indicate staining at cell-cell junctions. Scale bar = 10 µm. (**C**) 293VnR cells were transiently transfected with ADAM12-GFP WT or ADAM12-*myc p.L792F* and processed as described in B, except that cells were labeled with anti-myc instead of anti-ADAM12 primary antibody. Scale bar = 10 µm.

To evaluate expression and processing of ADAM12 *p.L792F*, we initially expressed ADAM12 WT and ADAM12 *p.L792F* mutant in 293VnR cells, which in contrast to many other cell lines readily express human full-length ADAM12 protein. ADAM12 *p.L792F* was expressed at the same levels as ADAM12 WT and, contrary to what has been described for the other two ADAM12 mutants [19], there was no difference in zymogen processing between WT and ADAM12 *p.L792F* (Fig. 2A). The similar molecular masses of the two major forms also suggested that there was no alteration of glycosylation. To assess if the two proteins exhibit similar intracellular localization, 293VnR cells transiently expressing WT or ADAM12 *p.L792F* were analyzed by immunofluorescence and confocal microscopy. Fixed and permeabilized cells were stained with the anti-ADAM12 monoclonal antibody 6E6. WT and mutant ADAM12 constructs had similar transfection efficiency and were expressed at similar levels. Both proteins localized to punctate intracellular organelles located in the perinuclear region (Fig. 2B left-hand images) and were also localized to cell-cell contacts (Fig. 2B right-hand images), indicating similar steady-state cellular distribution of the two proteins. Furthermore, when GFP-tagged ADAM12 WT was expressed in the same cell as a *myc-*tagged ADAM12 *p.L792F* there was complete co-localization of the two tags (Fig. 2C). This finding is in marked contrast to the two other cancer-associated mutations (*p.D301H* and *p.G479E*), which resulted in intracellular retention [19]. In many cases mutations in di-leucine trafficking motifs have severe consequences [20], EGFR being one example, where substitution of either of its two di-leucine motifs alters expression [22] and internalization [23]. However, our findings demonstrate that mutating the ADAM12 di-leucine motif had no obvious effect on expression level, processing, or overall cellular localization of the protein.

### ADAM12 *p.L792F* is internalized at the same level and rate as wild-type ADAM12

**Figure 3:**
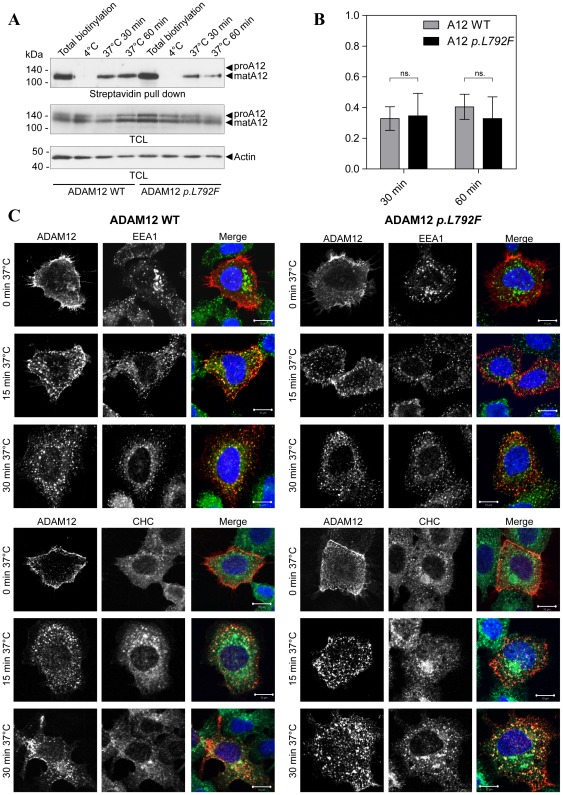
ADAM12 *p.L792F* localizes to the cell surface and is internalized similarly to wild-type ADAM12. (**A**) Biotinylation of cell surface ADAM12 WT or ADAM12 *p.L792F*. 293VnR cells, transiently transfected with ADAM12-GFP or ADAM12-GFP *p.L792F* were labeled with NHS-SS-Biotin for 30 min at 4°C, with subsequent incubation at 4°C (controls) or at 37°C for indicated time points. Then, the cells were treated with reducing reagent (glutathione) to remove non-internalized biotin from the cell surface (except for total biotinylation control lane 1). The control samples kept at 4°C were included to ensure proper removal of cell surface biotin. Biotinylated proteins were precipitated with streptavidin-conjugated agarose beads and subjected to western blotting with the appropriate antibodies. The ADAM12-GFP proform (proA12) is ∼150 kDa and the mature form (matA12) is ∼130 kDa. Actin was used as a loading control. TCL: Total cell lysate. (**B**) Quantitative analysis of ADAM12 internalization where internalized ADAM12-GFP at 30 or 60 min at 37°C were calculated as fold-change compared to total biotinylated ADAM12-GFP for each WT or mutant sample. The data are shown as +/− standard error of the mean (SEM) (n = 5 independent experiments); “ns.” indicates no statistical difference (p>0.05, two-tailed unpaired t-test). (**C**) ADAM12 WT or ADAM12 *p.L792F* internalization and co-localization with the early endosomal marker (EEA1) and clathrin heavy chain (CHC). The images show immunofluorescent staining and confocal laser-scanning microscopy of 293VnR cells transiently transfected with ADAM12 WT or the *p.L792F* mutant and seeded onto FBS-coated coverslips. Cells were labeled with ADAM12 monoclonal antibody 6E6 for 1 hour at 4°C, washed, and incubated in culture medium for 15 min or 30 min, followed by fixation and permeabilization. Cells were then stained with an EEA1 antibody (top three panels) or CHC antibody (bottom three panels) for 1 h at RT and subsequently with appropriate secondary antibodies and DAPI (n = 2). Merged images show partial co-localization of ADAM12 and EEA1 or CHC. Scale bar = 10 µm.

Since the di-leucine motif could facilitate endocytosis from the cell surface [20], [21], we compared the internalization of WT and ADAM12 *p.L792F*. A cell surface biotinylation assay, using cleavable biotin was used to determine the amounts of internalized protein. In this assay, cell surface-associated biotin, which has not been internalized, can be removed by washing with reducing agent. Transiently transfected 293VnR cells expressing ADAM12 WT or the *p.L792F* mutant were biotinylated at 4°C and proteins were allowed to be internalized for 30 or 60 min at 37°C. Total cell surface levels of either WT ADAM12 or ADAM12 *p.L792F* were determined in cells kept at 4°C and not treated with reducing agent. While some variability in the amount of cell surface protein was observed, quantitative analysis of independent replicate experiments did not disclose any difference in the total amount of ADAM12 WT and ADAM12 *p.L792F* proteins on the cell surface (Fig. 3A (1∶1.03 n = 5)). Moreover, incubation at 37°C for 30 or 60 min showed similar levels and rates of internalization between ADAM12 WT and ADAM12 *p.L792F* (Fig. 3A,B). Internalization and subsequent intracellular localization of ADAM12 *p.L792F* was evaluated by immunofluorescent staining and confocal microscopy. ADAM12 WT or ADAM12 *p.L792F* expressing cells were labeled with the anti-ADAM12 antibody 6E6 at 4°C to detect only cell surface proteins. Cells were then either fixed immediately (0 min) or incubated for 15 or 30 min at 37°C to allow protein internalization followed by fixation. Previous publications [24], [25] as well as our own unpublished data (Stautz et al, manuscript in revision) suggests that ADAM12, and possibly other ADAMs are internalized via the clathrin-dependent pathway and that after initial internalization ADAM12 localizes to early endosomes. We therefore included clathrin heavy chain (CHC) and early endosomal antigen 1 (EEA1) as markers in these stainings, to evaluate if the ADAM12 *p.L792F* behaved differently. At 0 min, both ADAM12 WT and ADAM12 *p.L792F* displayed cell surface staining, confirming the presence of both proteins at the cell surface (Fig. 3C, top row). After 15 or 30 min incubation at 37°C, both WT ADAM12 and ADAM12 *p.L792F* could be seen in punctate intracellular structures, and at all time-points there was partial co-localization with EEA1 and CHC (merged images, Fig. 3C), indicating that the same internalization pathway(s) is/are employed, and that once internalized the proteins localize similarly. After 15 min of incubation at 37°C, both proteins localized to punctate structures mainly close to the cell surface, whereas after 60 min of incubation at 37°C, localization was predominantly perinuclear. Together these data suggest similar temporal and spatial dynamics of WT ADAM12 and ADAM *p.L792F* during internalization. We conclude that the mutation did not obviously affect ADAM12 trafficking.

### The *p.L792F* mutation does not alter ADAM12-mediated cell proliferation or proteolytic activity

**Figure 4:**
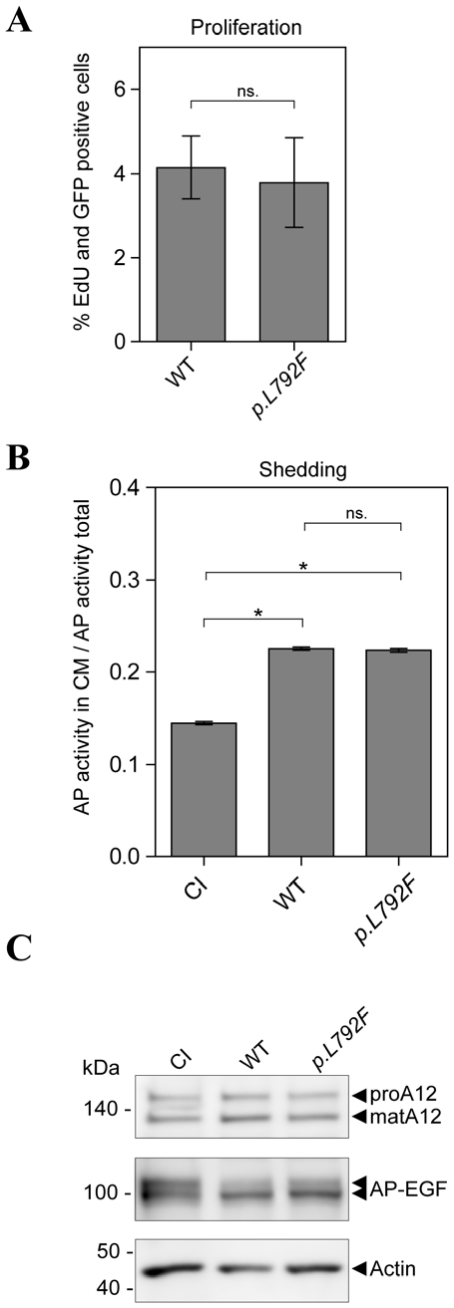
ADAM12 *p.L792F* does not affect cell proliferation or EGF shedding. (**A**) Comparable proliferation of ADAM12 WT and ADAM12 *p.L792F* expressing cells. 293VnR cells transiently transfected with ADAM12-GFP or ADAM12-GFP *p.L792F* were plated on FBS-coated coverslips in triplicates. Proliferating cells were stained with Edu. Four images were taken from each triplicate and EdU- and GFP-positive cells were calculated as percentage of total cells (DAPI staining). The data are shown as +/− standard error of the mean (SEM) (n = 2 independent experiments performed in triplicates); “ns.” indicates no statistical difference (p>0.05, two-tailed unpaired t-test). (**B**) ADAM12 *p.L792F* does not affect ADAM12-mediated proEGF shedding. 293VnR cells were transiently transfected with alkaline phosphatase (AP)-tagged proEGF together with ADAM12-GFP WT, CI ADAM12-GFP, or ADAM12 *p.L792F* (*p.L792F*). EGF-shedding was calculated from the ratio of AP activity in the cell media to total AP activity in the media and corresponding cell lysate, corrected for background (sample without AP substrate) (n = 2 independent experiments performed in triplicate). * indicates a statistically significant difference (p<0.0001, two-tailed unpaired t-test) and “ns.” indicates no statistical difference (p>0.05). (**C**) Representative western blot of protein expression control from experiment in (**B**). Transfected 293VnR cells were lysed in RIPA buffer and analyzed by western blot with the appropriate antibodies. Note the reduction in the upper band of AP-EGF, indicating shedding of proEGF-AP from the cell surface. Actin was used as a loading control.

We evaluated two key potential biological consequences of the *p.L792F* mutation. Since recent findings showed an effect of ADAM12 on tumor cell proliferation [13], ADAM12 *p.L792F* was tested for effects on cell proliferation. However, in analysis of cells expressing WT or ADAM12 *p.L792F* by EdU incorporation, no difference in the number of dividing cells was seen (Fig. 4A). Since ADAM12 is a catalytically active ADAM, we investigated if the *p.L792F* mutation affected ADAM12-mediated shedding of pro-epidermal growth factor (proEGF). Cells expressing alkaline phosphatase-tagged proEGF (AP-EGF) together with catalytically inactive (CI) ADAM12, ADAM12 WT, or ADAM12 *p.L792F* were tested for release of AP-EGF into the culture medium. All proteins were expressed at similar levels (Fig. 4C) and both ADAM12 WT and ADAM12 *p.L792F* showed increased shedding of proEGF compared to CI ADAM12. However, ADAM12 *p.L792F* -mediated shedding did not differ from that of ADAM12 WT (Fig. 4B). Thus, based on these data, the *p.L792F* mutant has no apparent functional impact in the assays employed.

In summary, we have investigated one of three previously reported breast cancer-associated mutations in the metalloprotease ADAM12, namely the intracellular *p.L792F* mutation. Despite the affected residue being situated in a potential di-leucine trafficking motif, evaluation of the mutant protein did not reveal any detectable effects on expression, processing, trafficking or known ADAM12 function, as compared to ADAM12 WT. This lack of a functional effect is consistent with leucine 792 not being conserved in human and mouse ADAM12, and indeed, few human ADAMs contain di-leucine motifs (LL or LI), arguing against this being a general trafficking signal for ADAM proteins. ADAM12 has a second di-leucine motif upstream of the one currently investigated, which is more conserved between species. Yet, this sequence deviates more from consensus di-leucine trafficking motifs. Interestingly, a non-consensus di-leucine motif is also found in the related transmembrane MT1-MMP and this seems to be important for protein glycosylation, rather than protein trafficking [26]. However, no impact of the mutation on ADAM12 glycosylation was observed. Thus, based on the reported data, we suggest that the *p.L972F* mutation in ADAM12 is likely a passenger mutation and not a cancer causing driver mutation.

## Methods

Unless otherwise stated, chemicals were purchased from Sigma-Aldrich (St. Louis, MO, USA).

### Antibodies

Mouse monoclonal antibody 6E6 directed against the disintegrin and cysteine-rich domains of ADAM12 was previously described [27]. Primary antibodies: ADAM12 Polyclonal Antibody (ProteinTech Group, Chicago, IL, USA), GFP antibody full-length a.v. polyclonal antibody (Clontech, Mountain View, CA, USA), mouse anti-actin and anti-myc monoclonal antibodies (Millipore, Bedford, MA, USA), rabbit polyclonal anti-EEA1 and anti-CHC antibodies (Abcam, Cambridge, UK). Secondary antibodies for western blot were polyclonal goat anti-mouse immunoglobulins/HRP and polyclonal goat anti-rabbit immunoglobulins/HRP (Dako A/S, Glostrup, Denmark). Secondary antibodies for immunofluorescent staining were Alexa Fluor® 546 F(ab′)_2_ fragment of goat anti-mouse IgG and Alexa Fluor® 546 F(ab′)_2_ fragment of goat anti-rabbit IgG (Invitrogen, Carlsbad, CA, USA).

### Cell culture

Human epithelial HEK293VnR cells stably expressing the vitronectin receptor (αvβ3 integrins) (henceforth referred to as 293VnR) [28] were maintained in Dulbecco's Modified Eagle Medium (DMEM) with GlutaMAX™-I, supplemented with 10% Fetal Bovine Serum (Hyclone, Perbio, Bonn, Germany).

### Expression constructs

Mammalian expression constructs for full-length membrane-bound human ADAM12 (ADAM12-L), human ADAM12 fused at its C-terminus to green fluorescent protein (ADAM12-GFP) and catalytically inactive (CI) ADAM12-GFP (E351Q mutation) were previously described [29], [30]. ADAM12-*myc*-His was made by excision of ADAM12 cDNA from the pEGFP-N1 vector using *XhoI* and *BamHI* restriction enzymes and ligation into the corresponding pcDNA3.1/*myc*-His© B vector (Invitrogen). The leucine to phenylalanine (*p.L972F*) mutation in ADAM12, ADAM12-*myc* and ADAM12-GFP were made by Mutagenex, Innovative Mutagenesis and Directed Evolution (www.mutagenex.com). ProEGF fused to alkaline phosphatase (AP) in the pRC/CMV expression vector (proEGF-AP) was kindly provided by Dr. S. Higashiyama, Ehime University Graduate School of Medicine, Ehime, Japan.

### Transfections

293VnR cells were transfected using OPTI-MEM® I Reduced Serum Medium (Gibco, Invitrogen) and FuGENE® 6 Transfection Reagent (Roche Applied Science, Hvidovre, Denmark) according to manufacturer's instructions. Experiments were carried out 48 h after transfection.

### Cell surface biotinylation assay

All reagents were kept at 4°C and biotinylation with membrane impermeable reagents was carried out at 4°C to selectively label cell surface proteins. Transiently transfected 293VnR cells were preincubated at 4°C for 15 min to stop ongoing internalization, washed in cold phosphate buffered saline (PBS, Invitrogen), and incubated for 30 min at 4°C with 0.05 mg/ml EZ-Link™ Sulfo-NHS-SS-Biotin (Pierce, Thermo Scientific, Rockford, IL, USA) in cold PBS. Biotinylation was quenched by three washes in 100 mM glycine (AppliChem, Darmstadt, Germany) in cold PBS and once in cold serum-free DMEM. Fresh cold serum-free DMEM was added to the cells, which, except for the controls were transferred to 37°C for the time points required for each experiment. After incubation, cells were washed once in cold PBS and non-internalized cell surface biotin was removed by washing 3×10 min in stripping buffer (50 mM L-Glutathione, 75 mM NaCl, 75 mM NaOH, 1% (w/v) bovine serum albumin (BSA) and 10 mM ethylenediaminetetraacetic acid (EDTA) pH 8.0). Finally, cells were washed three times in cold PBS and lysed in radioimmunoprecipitation assay (RIPA) buffer (50 mM Tris pH 7.4; 150 mM NaCl; 0.1% sodium dodecyl sulphate (SDS); 1% Triton X-100 0.5% sodium deoxycholate; 1 mM EDTA, and complete EDTA-free inhibitor cocktail (Roche)). Cell lysates were cleared by centrifugation for 20 min at 16,000 g and supernatants were incubated with streptavidin-agarose (Sigma) for 2 h at 4°C. Beads were washed three times in RIPA buffer and bound proteins were released by heating for 5 min at 95°C in 2× SDS-PAGE sample buffer (0.0625 M Tris pH 6.8, 20% glycerol, 2% SDS, 0.01% bromophenol blue, 5% 2-mercaptoethanol). Samples were analyzed by western blot.

### Western blotting

Total cell lysates and streptavidin affinity-isolated proteins were subjected to standard 7.5% or 10% reducing SDS-PAGE and transferred to Hybond-ECL nitrocellulose membranes (GE Healthcare). The membranes were incubated with the indicated primary antibodies and subsequently with the appropriate horseradish peroxidase (HRP)-conjugated secondary antibody. The EZ ECL Chemiluminescence Detection Kit for HRP (Biological Industries Ltd, Kibbutz Beit Haemek, Israel) was used to detect antibody binding and images were acquired using the LAS3000 Imager (Fujifilm), or using Hyperfilm ECL high performance chemiluminescence film (GE Healthcare) and a Valsoe X-Ray developer (Ferrania, Valsoe X-ray, Hoejbjerg, Denmark).

### Densitometric analysis of western blots

The TotalLab TL100 software (TotalLab Limited) was used to perform densitometric analysis. Briefly, total cell surface biotinylated ADAM12, biotinylated endocytosed ADAM12, and ADAM12 in the total cell lysates (TCL/input) were quantified in unsaturated (brief exposure) western blot images (mature ADAM12 form only). Levels of ADAM12 in the pull-down assay were adjusted to ADAM12 levels in the total cell lysate for each WT or mutant protein and then the fold-endocytosed ADAM12 was calculated from the total cell surface biotinylated ADAM12. In each case, total cell surface biotinylated ADAM12 was set to 1.

### Immunofluorescence and confocal microscopy

Transiently transfected 293VnR cells were plated on FBS-coated coverslips (Menzel, Braunschweig, Germany) and allowed to attach and spread for 24 h. Cells were then either fixed directly in 4% paraformaldehyde (PFA, Merck, Darmstadt, Germany) at room temperature (RT) (for permeabilized cell staining) or, for cell surface staining, preincubated at 4°C for 15 min to stop ongoing internalization, and subsequently incubated with 6E6 antibody diluted in 1% BSA in PBS for 1 h at 4°C. Cells were washed in cold PBS and cold DMEM was added to the cells, which, except for the controls, were transferred to 37°C at the time points indicated. After incubation, cells were fixed in fresh 4% PFA at RT and permeabilized in 0.5% Triton X-100/PBS. Free aldehyde groups were quenched with 0.1 M NH_4_Cl/PBS and cells were blocked with 5% BSA/PBS. For co-staining with 6E6 and EEA1, cells were incubated with primary antibody diluted in 1% BSA in PBS for 1 h at RT. All cells were stained with the appropriate Alexa 488 or Alexa 546 secondary antibodies and 4′,6-diamidino-2-phenylindole (DAPI) (Invitrogen) in 1% BSA/PBS for 1 h at RT. Coverslips were mounted on Super Premium Microscope Slides (VWR, Albertslund, Denmark) using DAKO® Faramount Aqueous Mounting Medium (Dako). Micrographs were acquired on a confocal microscope (Axiovert 200 M LSM 520; Carl Zeiss, Inc.) using a 63× C-Apochromat objective with a numerical aperture of 1.2 in H_2_O. Micrographs were taken at room temperature. The images were processed using the LSM Image Browser software (Carl Zeiss, Inc.).

### Cell proliferation assay

293VnR cells transiently transfected with ADAM12-GFP WT or ADAM12-GFP *p.L972F* were plated in triplicate on FBS-coated coverslips and allowed to attach and spread for 24 h. Proliferating cells were stained using the Click-iT® EdU Alexa Fluor® 594 Imaging Kit (Invitrogen) according to manufacturer's instructions and nuclei were stained using DAPI. Four images from each triplicate experiment were acquired using the Zeiss Axiovert 200 M ApoTome (Carl Zeiss, Inc.) with Axiovision software and analyzed with the Metamorph software. Nuclei positive for both EdU and GFP were calculated as percentage of total nuclei (DAPI stain).

### Shedding assay

293VnR cells were transiently transfected with proEGF-AP together with WT ADAM12-GFP, catalytically inactive ADAM12-GFP, or ADAM12-GFP *p.L972F*. Forty-eight hours after transfection, each transfection was split into four wells in a 24-well plate. The next day, one well of each transfection was washed 3× in PBS and lyzed in RIPA buffer to serve as an expression control in analysis by Western blot. The remaining wells (triplicates) were washed twice with PBS, followed by 2 h incubation with fresh serum free medium. For photometric quantitation of proEGF-AP shedding, cell-conditioned medium was harvested and the cell layer lysed in 1% Triton X-100 in PBS. Conditioned serum free medium or cell lysate (50 µl) from each well was mixed with 50 µl of a 2 mg/ml solution of the alkaline phosphatase substrate 4-nitrophenyl (Sigma), with each well assayed in duplicate in a 96-well plate. Alkaline phosphatase activity was quantified as absorbance at 405 nm as a function of time, and the fold-change in EGF-shedding activity was calculated as alkaline phosphatase activity in the conditioned media divided by total alkaline phosphatase activity in the media plus the corresponding cell lysate. CI ADAM12 was set as 1 and fold shedding by the corresponding ADAM12 WT and mutant was calculated.
